# Performance Optimization of Lead-Free Cs_2_AgBiI_6_ Solar Cells with the LBSO Electron Transport Layer: A SCAPS-1D Simulation Study

**DOI:** 10.1021/acsomega.6c06922

**Published:** 2026-09-26

**Authors:** Ragulraj Premkumar, Thangaraji Vasudevan, Hariharan Rajasekaran, Lung-Chien Chen

**Affiliations:** Department of Electro-Optical Engineering, 34877 National Taipei University of Technology, Taipei City 10608, Taiwan (R.O.C)

## Abstract

Lead toxicity in conventional halide perovskites has long been a concern for practical photovoltaic deployment, and Cs_2_AgBiI_6_ double perovskites gained significant attention as promising lead-free alternatives due to their high carrier mobility, suitable optoelectronic characteristics, and relatively good chemical stability. In this work, density functional theory (DFT) calculations were performed to investigate the electronic properties of the Cs_2_AgBiI_6_ absorber, while SCAPS-1D simulations were independently employed to optimize the photovoltaic performance of the proposed device architecture, using lanthanum-doped barium stannate (LBSO) as the electron transport layer (ETL), which, to our knowledge, has not been previously reported for this absorber. LBSO was compared against CdS and ZnS across thicknesses of 10–100 nm, while six HTL candidates, including CBTS, Cu_2_O, CuSCN, CuI, Spiro-MeOTAD, and PEDOT:PSS, were evaluated simultaneously. Interface trap densities at both junctions were swept from 1 × 10^9^ to 1 × 10^20^ cm^–3^, revealing that the HTL-side interface is significantly more recombination-sensitive than the ETL side, which DFT analysis helped explain through electronic structure and defect formation energy calculations. Absorber bulk defect density above ∼10^13^ cm^–3^ was found to severely degrade performance, and absorber thickness was optimized between 100 and 1200 nm. The final optimized FTO/LBSO/Cs_2_AgBiI_6_/Cu_2_O/Au structure with achieved *J*_sc_ =24.66 mA/cm^2^, *V*_oc_ =1.248 V, PCE=27.52%, and FF = 89.42% under AM1.5G illumination, demonstrating the potential of the optimized device architecture for lead-free double perovskite photovoltaics.

## Graphical abstract

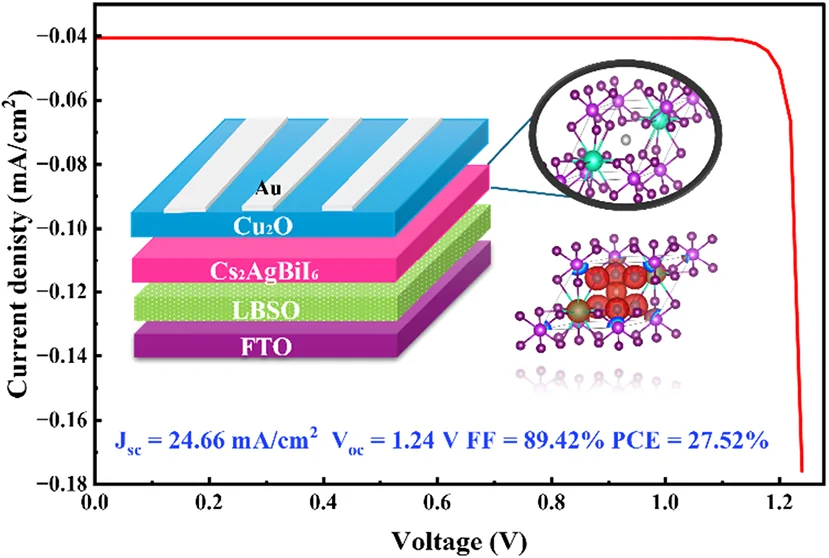

## 1 Introduction

Perovskite photovoltaic cells have attracted significant interest in recent years, due to their superior light-absorption properties, tunable bandgap, long carrier diffusion lengths, and enhanced tolerance toward defects.^1,5^ Beginning with an initial efficiency of only 3.8% in the first reported device,^4^ certified efficiencies have now crossed 27% for single-junction cells and exceeded 30% in tandem configurations.^2,3^ These devices can also be made using vapor-based deposition methods and low-temperature solution, which makes them an attractive option for cost-effective and scalable photovoltaic manufacturing. The excellent performance and simple device fabrication of perovskite solar cells (PSCs) have made them strong contenders for next-generation renewable energy technologies.

Two persistent problems, however, continue to hold back the widespread use of conventional lead-based perovskites. The presence of Pb^2+^ raises genuine environmental concerns, particularly around leakage during device aging or end-of-life disposal, with documented risks of aqueous dissolution and accumulation in biological systems.^6,7^ Regulatory pressure from frameworks such as the EU RoHS Directive has added further urgency to the search for lead-free alternatives in electronic and energy devices.^9^ At the same time, organic A-site cations like methylammonium and formamidinium tend to break down under heat, moisture, and sustained illumination, which shortens device lifetimes and limits their practical use in real outdoor conditions.^8^ Together, these two issues have pushed researchers to look for materials that can keep the good electronic properties of perovskites while doing away with both the toxicity and the stability concerns.

Among the lead-free candidates that have emerged, vacancy-ordered double perovskites have attracted growing interest for their ability to completely remove lead while preserving the corner-sharing octahedral framework.^10,11^ Cs_2_AgBiI_6_ stands out within this group because its fully inorganic cesium A-site makes it considerably greater resistant to thermal stress and moisture-related degradation compared with organic-cation-based counterparts, and its iodide sublattice brings the bandgap down to around 1.6 eV a value well positioned for harvesting visible light and notably better than what bromide or chloride double perovskite compositions can offer.^12–14^

Getting high efficiency out of Cs_2_AgBiI_6_ devices, however, is not straightforward. The transport layers on either side of the absorber have a strong influence on overall device output, since mismatched band alignment at these interfaces tends to create energy barriers and push up recombination losses.^15,16,20^ TiO_2_, the most widely used ETL, is held back by its low electron mobility and tendency to degrade under UV exposure,^17,18,37^ which has driven growing interest in La-doped BaSnO_3_ (LBSO) as a more stable and higher-mobility option.^19,38^ On the hole-transport side, materials such as Cu_2_O, CuSCN, and CuI have attracted considerable interest as thermally stable alternatives to hygroscopic organic HTLs, though how well each of them lines up energetically with Cs_2_AgBiI_6_ varies quite a bit and needs to be properly evaluated.^21–23^ Most published simulation work on this absorber, however, has looked at individual parameters one at a time rather than putting together a full and connected device optimization.

This work addresses that gap by combining SCAPS-1D simulations with DFT calculations to screen and optimize transport layer materials, layer thicknesses, and interface defect densities for Cs_2_AgBiI_6_-based solar cells in one coordinated study. LBSO is used as the ETL for the first time for this absorber and consistently delivers the higher performance, while Cu_2_O is found as the optimal HTL based on its favorable band alignment and low rear interface recombination. The fully optimized FTO/LBSO/Cs_2_AgBiI_6_/Cu_2_O/Au device delivers a PCE of 27.52%, along with a Voc of 1.248 V, Jsc of 24.66 mA/cm^2^, and FF of 89.42% under AM1.5G illumination, providing valuable theoretical guidance for the future design and optimization of lead-free double perovskite photovoltaics.

## 2 Device Architecture and Simulation Methodology

### 2.1 SCAPS-1D Simulation Platform

The device characteristics of the proposed Cs_2_AgBiI_6_-based solar cell was analysed using one-dimensional simulations in SCAPS-1D (version 3.3.10), created at Ghent University.^24,39^ The software iteratively solves the coupled Poisson and carrier continuity equations to obtain the electrostatic potential, carrier concentrations and current density under steady-state conditions. Charge transport in each layer occurs through both drifts, driven by the internal electric field, and diffusion, driven by carrier concentration gradients, with their relationship governed by the Einstein relation. Optical generation was calculated under standard AM1.5G solar illumination at 100 mW/cm^2^ using material-specific absorption coefficients, while reflection losses were neglected. Simulations were performed at 300 K with ohmic contacts assumed at the FTO front electrode and Au back contact. The simulation model incorporated charge-carrier loss mechanisms associated with Shockley–Read–Hall (SRH), radiative, and non-radiative Auger recombination, with SRH recombination considered the dominant loss pathway. The absorber defect density (*N*_t_) was considered a key parameter in the study. To examine the impact of interface quality on device behaviour, interfacial recombination at both the ETL/absorber and absorber/HTL contacts was incorporated through the use of interface defect densities.

The material parameters for each device layer were obtained from earlier theoretical reports and are listed in Tables 1 and 2. For the Cs_2_AgBiI_6_ absorber, a single reference source was used to maintain consistency across all input values bandgap 1.6 eV, electron affinity 4.05 eV, relative permittivity 6.5, electron and hole mobilities 20 cm^2^ V^–1^ s^–1^, band edge densities of states 1 × 10^19^ cm^–3^, donor density 1 × 10^17^ cm^–3^, and bulk defect density 1 × 10^14^ cm^–3^. For LBSO, the electron affinity was set to 4.4 eV to correctly capture the conduction band offset at the front junction.^25^ The remaining ETL and HTL parameters are detailed in Tables 1 and 2.^26^ Throughout all layers, bulk defects were represented as neutral mid-gap traps with capture cross sections of 1 × 10^–15^ cm^2^. At both device junctions, interface defect states were given an integrated density of 1 × 10^10^ cm^–2^, with the full capture cross-section details provided in Table 3. The SCAPS-1D simulations were performed using the default electrical boundary conditions (*R*_s_ = 0 Ω·cm^2^ and *R*_sh_ = ∞), representing an idealized device without parasitic resistive losses. For the Cs_2_AgBiI_6_ absorber, the reported parameters were adopted from reference^26^ to maintain consistency across the input set, and their values were cross-checked with other reported studies.

**Table 1:** Input Parameters of HTL Layer

**parameters**	**CBTS**	**Cu**_**2**_ **O**	**Spiro-MeOTAD**	**CuSCN**	**CuI**	**PEDOT: PSS**
thickness (nm)	100	50	200	50	100	50
band gap, Eg (eV)	1.9	2.2	3	3.6	3.1	1.6
electron affinity, (eV)	3.6	3.4	2.2	1.7	2.1	3.5
dielectric permittivity, ε_r_	5.4	7.5	3	10	6.5	3
CB effective density of states, *N*_c_ (1/cm^3^)	2.2 ×10^18^	2 × 10^19^	2.2 × 10^18^	2.2 × 10^19^	2.8 ×10^19^	2 × 10^21^
VB effective density of states, *N*_v_ (1/cm^3^)	1.8 × 10^19^	1 × 10^19^	1.8 × 10^19^	1.8 × 10^18^	1 ×10^19^	2 ×10^21^
electron mobility, μ_n_ (cm^2^/(V s))	30	200	2.1 ×10^–3^	100	100	1.8 ×10^–3^
hole mobility, μ_h_ (cm^2^/(V s))	10	8600	2.16 ×10^–3^	25	43.9	1.86 ×10^–2^
shallow uniform acceptor density, *N*_A_(1/cm^3^)	1 × 10^18^	1 × 10^18^	1 × 10^18^	1 × 10^18^	1 × 10^18^	1 × 10^18^
shallow uniform donor density, *N*_D_ (1/cm^3^)	0	0	0	0	0	0
defect density, *N*_t_ (1/cm^3^)	1 × 10^15^	1 × 10^15^	1 ×10^15^	1 × 10^15^	1 × 10^15^	1 × 10^15^

**Table 2:** Simulation Parameters for the ETL, FTO, and Absorber Layers

**parameters**	**CdS**	**ZnS**	**LBSO**	**Cs**_**2**_ **AgBiI**_**6**_	**FTO**
thickness (nm)	30	50	100	1200	200
band gap, *E*_g_ (eV)	2.4	3.6	3.12	1.6	3.5
electron affinity, (eV)	4.18	3.5	4.4	4.05	4
dielectric permittivity, ε_r_	10	8.9	22	6.5	9
CB effective density of states, *N*_c_(1/cm^3^)	1 × 10^18^	1.8 × 10^19^	1.8 × 10^20^	1 × 10^19^	2.2 ×10^18^
VB effective density of states, *N*_v_ (1/cm^3^)	1.9 × 10^19^	2.4 × 10^18^	1.8 × 10^20^	1 × 10^19^	1.8 ×10^19^
electron mobility, μ_n_ (cm^2^/(V s))	100	120	69 ×10^–2^	20	20
hole mobility, μ_h_ (cm^2^/(V s))	43.9	10	69 ×10^–2^	20	10
shallow uniform acceptor density, *N*_A_(1/cm^3^)	0	0	0	0	0
shallow uniform donor density, *N*_D_ (1/cm^3^)	1 × 10^18^	1 × 10^18^	1 × 10^18^	1 × 10^17^	1 × 10^18^
defect density, *N*_t_ (1/cm^3^)	1 × 10^15^	1 × 10^15^	1 ×10^15^	1 × 10^14^	1 × 10^15^

**Table 3:** SCAPS-1D Interface Defect Parameters for Cu_2_O/Cs_2_AgBiI_6_ and Cs_2_AgBiI_6_/LBSO Junctions^a^

**interface**	**defect charge state**	**electron capture section(cm** ^**2**^ **)**	**hole capture section(cm** ^**2**^ **)**	**energetic distribution**	**defect energy level reference**	**integrated defect density(cm** ^**‑2**^ **)**
Cu_2_O/Cs_2_AgBiI_6_	neutral	1 × 10^–17^	1 × 10^–18^	*m*	*n*	1 × 10^10^
Cs_2_AgBiI_6_/LBSO	neutral	1 × 10^–18^	1 × 10^–19^	*m*	*n*	1 × 10^10^

a ^*m*^ Single, ^*n*^ above the highest EV (**Note:** Bulk defect density *N*_t_ is expressed in cm^–3^, whereas interface defect density is expressed in cm^–2^, reflecting their volume- and area-based definitions, respectively.)

### 2.2 Density Functional Theory (DFT) Calculations

To obtain material-specific properties of the Cs_2_AgBiI_6_ absorber, first-principles calculations were carried out using Quantum ESPRESSO (v7.4.1). The simulations were carried out using DFT within the GGA (generalized gradient approximation), employing the PBE (Perdew–Burke–Ernzerhof) functional.^27,28^ Norm-conserving pseudopotentials were used for all elements (Cs, Ag, Bi, and I), Based on the convergence analysis, a plane-wave kinetic energy cutoff of 50 Ry, a charge-density cutoff of 500 Ry, and a 3 × 3 × 3 Monkhorst–Pack k-point mesh were adopted for all calculations. The crystal structure was fully relaxed until the forces on atoms and total energy reached stable convergence.

The converged ground-state structure served as the basis for extracting the Kohn-Sham band dispersion, total and site/orbital-decomposed density of states (DOS/PDOS), and the all-electron electrostatic potential, providing a complete picture of the electronic and bonding characteristics of Cs_2_AgBiI_6_. These results provide a clear picture of the electronic behaviour and band characteristics of Cs_2_AgBiI_6_.

The DFT calculations were performed to investigate the intrinsic electronic properties of Cs_2_AgBiI_6_ and to support the discussion of the simulation results. All input parameters used in the SCAPS-1D simulations were adopted from previously reported literature; therefore, the DFT calculations were not directly used to generate the SCAPS-1D input parameters. The present DFT calculations were performed without including spin–orbit coupling (SOC). While SOC is expected to improve the quantitative description of the electronic structure of Bi-based double perovskites, the objective of this study was to obtain qualitative insight into the electronic properties of Cs_2_AgBiI_6_. Therefore, the omission of SOC does not affect the qualitative interpretation presented in this work.

## 3 Results and Discussion

### 3.1 Band Alignment and Built-in Potential Analysis

The device schematic and band diagram of the FTO/LBSO/Cs_2_AgBiI_6_/Cu_2_O/Au structure are shown in Figure 1a,b. The FTO front contact, with a bandgap of 3.5 eV, acts as the transparent electrode where light enters the device. At the LBSO/Cs_2_AgBiI_6_ junction, a spike-type conduction band (CB) offset of 0.35 eV is formed due to the difference in electron affinity (EA) between LBSO (3.12 eV bandgap) from flowing back into the absorber. Inside the Cs_2_AgBiI_6_ absorber, both band edges tilt gradually across the 1200 nm layer, indicating a built-in electric field that helps separate and move carriers toward their respective contacts. At the rear Cs_2_AgBiI_6_/Cu_2_O junction, the conduction band drops from 4.05 to 3.4 eV, which keeps electrons away from the back contact. The valence band offset here is only around 0.05 eV, meaning holes can move into the Cu_2_O layer with very little energy barrier. From there, holes travel through the Cu_2_O HTL, which has a bandgap of 2.2 eV, and reach the Au back contact. The overall band alignment with a spike at the front and near-flat valence band at the rear supports efficient carrier collection at both junctions and is a key reason behind the high Voc of 1.248 V and PCE of 27.52% seen in this device.

**Figure 1:**
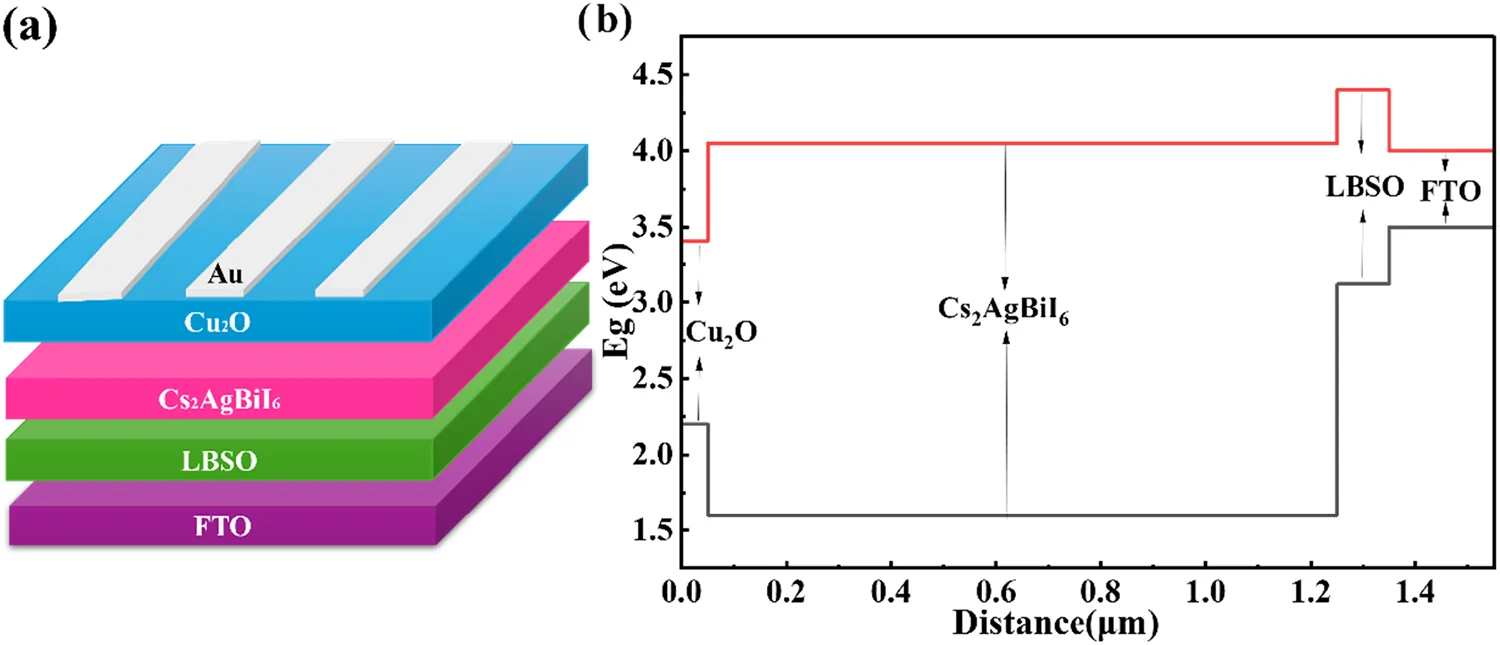
(a) Diagrammatic illustration of the multilayer device configuration and (b) the resultant energy band alignment across the constituent heterojunctions.

### 3.2 Electronic Band Structure of Cs_2_AgBiI_6_

The electronic band structure of Cs_2_AgBiI_6_ calculated along the high-symmetry k-path Γ→X→W→K→Γ→L→U→W→L→K→X of the face-centred cubic Brillouin zone is presented in Figure 2, with all energies referenced to the Fermi level at 0 eV.

**Figure 2:**
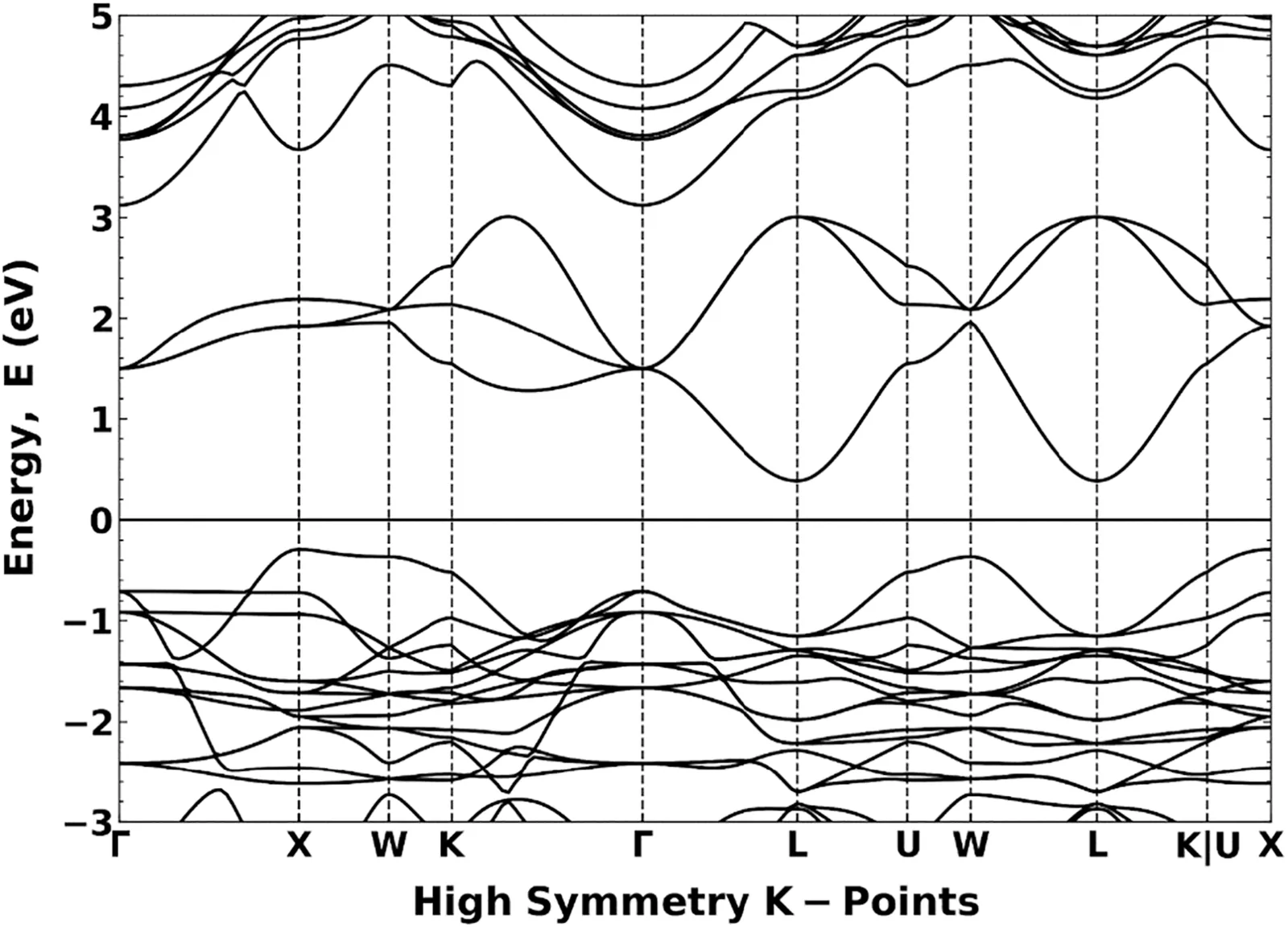
Calculated electronic band structures of Cs_2_AgBiI_6_.

The computed band profile establishes the semiconducting nature of the material through a well-defined energy gap of approximately 1.6 eV. The valence band ceiling is localized at the Γ point of the first Brillouin zone, whereas the conduction band floor is situated at a symmetry-inequivalent reciprocal lattice vector, yielding a momentum-forbidden fundamental transition that conclusively identifies Cs_2_AgBiI_6_ as an indirect-bandgap semiconductor. This finding is in consistent with previously reported GGA-PBE calculations for Ag–Bi-based double perovskite compositions.

Looking at the conduction band region just above the Fermi level, the lowest band shows reasonable dispersion near the Γ point before higher conduction bands spread over an energy range extending up to approximately 5 eV. This curvature at the CBM to relatively low electron effective masses, which is favorable for photogenerated electron transport toward the ETL. The valence band region between 0 and –3 eV, on the other hand, consists of several bands with noticeably flatter profiles, a clear indication of heavier hole effective masses. This asymmetry in band curvature between electrons and holes is a well-known characteristic of Ag–Bi vacancy-ordered double perovskites and is further supported by the projected density of states analysis discussed in the following section. The deeper valence states show multiple overlapping branches and band crossings that reflect the complex orbital interactions among Cs, Ag, Bi, and I in the lattice.

Taken together, the computed band structure confirms an indirect fundamental gap of 1.6 eV and highlights the inherent asymmetry in carrier transport within this material. It is worth noting that the PBE functional used in this work tends to underestimate bandgap values, and spin–orbit coupling was not included in the calculation, both of which may slightly affect the absolute band positions reported here. Previous theoretical studies have reported that SOC mainly modifies the quantitative band energies, whereas the indirect bandgap nature of Cs_2_AgBiI_6_ remains unchanged.

### 3.3 Projected Density of States (PDOS) Analysis of Cs_2_AgBiI_6_

Figure 3 shows the projected density of states (PDOS) of Cs_2_AgBiI_6_ that was found using first-principles calculations, with energies referenced to the Fermi level (FL) at 0 eV shown by the vertical dashed line. In the deep valence region between –15 and –10 eV, a series of sharp peaks arise predominantly from Cs and I state that are energetically far removed from the band edges. Since these states lie well below the frontier orbitals, they play no meaningful role in optical absorption or charge transport under photovoltaic operating conditions.

**Figure 3:**
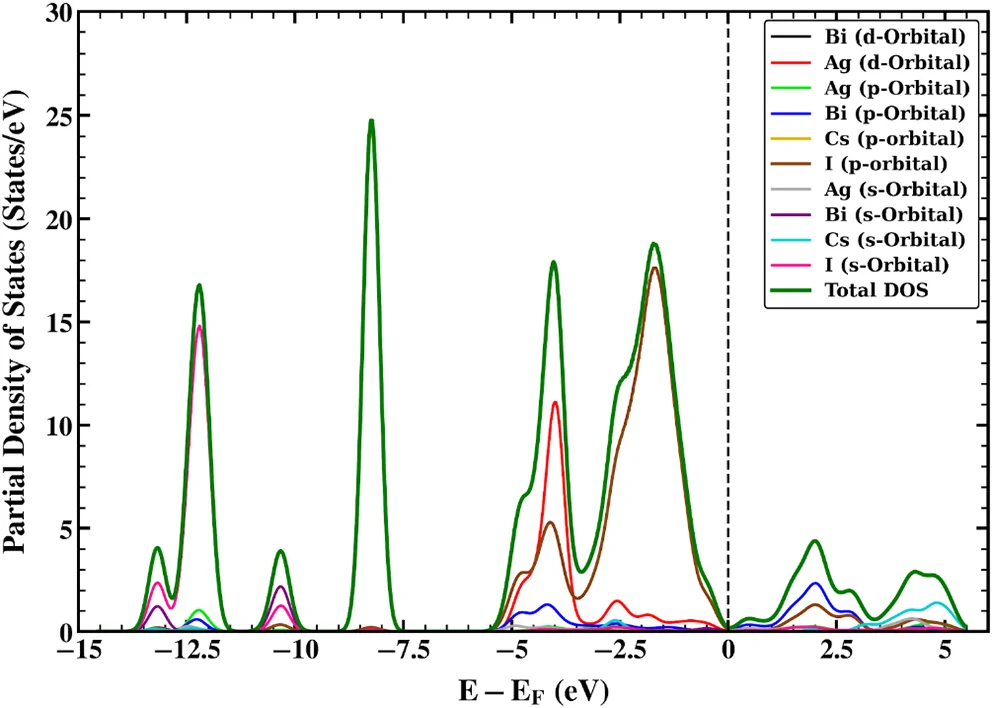
Calculated partial density of states (PDOS) for Cs_2_AgBiI_6_, revealing the orbital composition of the valence and conduction band edges. The dashed vertical line marks the Fermi level (FL).

Moving toward the VBM in the energy window from –6 to 0 eV, the spectral weight is mainly carried by a strong overlap between Ag d-orbitals and I p-orbitals, whose mutual interaction defines the character of the upper valence band. A smaller but visible Bi p-orbital contribution also appears near the band edge in this region. These features collectively indicate that photogenerated holes reside primarily on Ag–I bonding states, while Cs-derived contributions remain buried at deeper energies consistent with the purely structural spacer role of the Cs^+^ cation in the double perovskite lattice. Bi p-orbitals make up most of the available electronic states just above the Fermi level in the conduction band region. This makes Bi^3+^ the main electron-accepting species at the conduction band minimum. At higher conduction band energies, minor contributions from Bi s and Cs s states become visible, while Ag and I derived states remain comparatively weak throughout this region.

The PDOS analysis therefore reveals a well-defined orbital separation between the two band edges −Ag-I hybridized states at the VBM and Bi p-states at the conduction band minimum. This chemical distinction between the frontier states for holes and electrons is consistent with the asymmetric effective masses seen in the band structure and provides a clear orbital-level basis for understanding the differences in electron and hole transport behaviour observed in the SCAPS-1D device simulations.

### 3.4 Electrostatic Potential Analysis of Cs_2_AgBiI_6_

The electrostatic potential distribution of Cs_2_AgBiI_6_ is examined through the DFT-relaxed crystal structure and electron density iso-surface mapping presented in Figure 4, where Cs (teal), Bi (purple), Ag (grey), and I (dark purple) are identified as the four atomic species.

**Figure 4:**
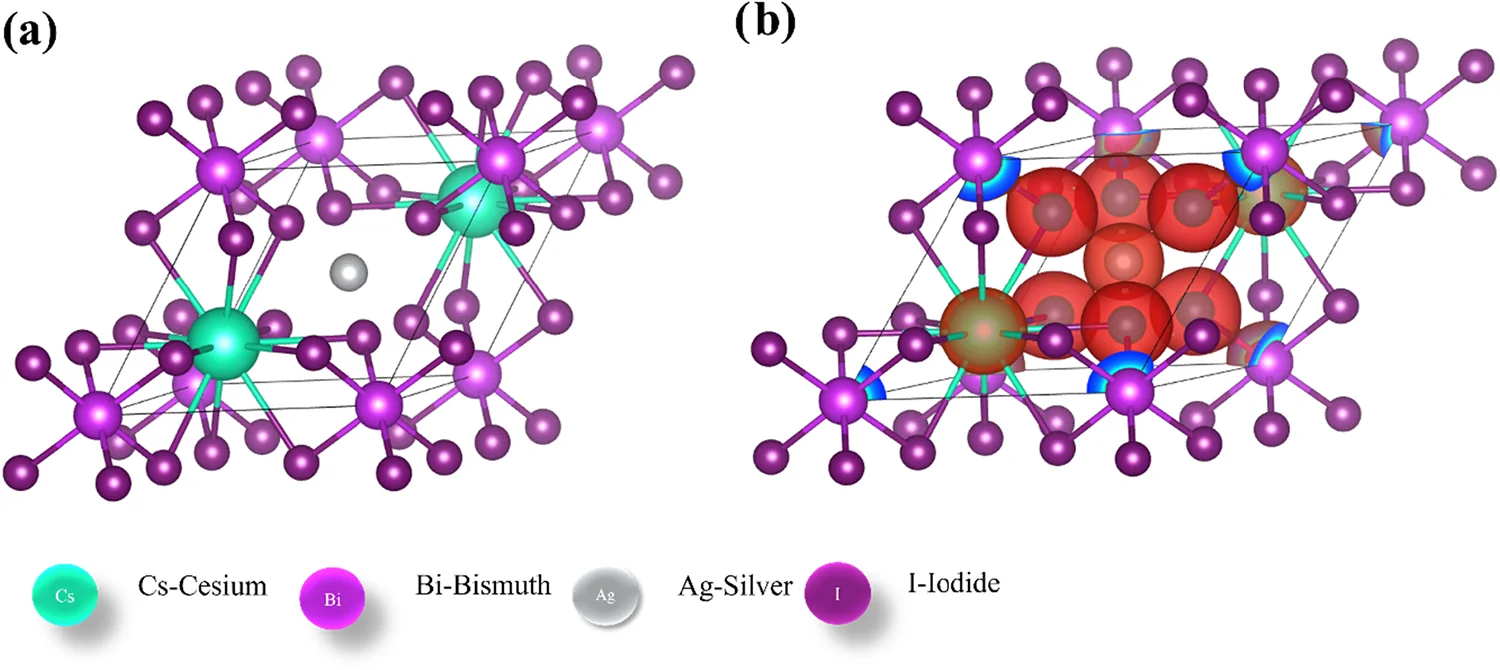
Optimized crystal structure (a) and electron density iso-surfaces (b) of Cs_2_AgBiI_6_ showing electrostatic potential distribution.

Figure 4a shows the fully relaxed crystal structure, confirming the rock-salt ordered double perovskite geometry in which Cs^+^ fills the A-site cub octahedral cavities, while Bi^3+^ and Ag^+^ alternate at the octahedrally coordinated B-sites within the iodide framework. The Cs^+^ ions are spatially confined to the lattice periphery and contribute primarily to structural stability, with negligible participation in the frontier electronic states a finding fully consistent with the deep positioning of Cs-derived states identified in the PDOS analysis. Figure 4b overlays the first-principles electron density iso-surfaces onto the same relaxed geometry. The charge density is found to be concentrated predominantly around the iodide anion sites, reflecting their electronegative character within the lattice, while the cation sites display comparatively lower electron density. This spatial charge distribution points to a predominantly ionic bonding environment with partial metal–halide orbital hybridization, in agreement with the Ag–I hybridized valence band character established by the PDOS.

The electrostatic potential variation across the lattice provides useful qualitative insight into the local bonding environment and charge distribution in Cs_2_AgBiI_6_. The deep and localized potential wells associated with the cation sites, separated by electrostatically neutral interstitial regions, are consistent with the indirect bandgap and asymmetric carrier effective masses obtained from the band structure calculations. These features collectively support the transport parameter choices adopted in the SCAPS-1D device simulations and help explain the sensitivity of device performance to bulk defect density observed in the optimization results.

### 3.5 Role of Cu_2_O/Cs_2_AgBiI_6_ Interface Defects in Determining Device Performance

The influence of interface defect concentration at the Cu_2_O/Cs_2_AgBiI_6_ rear junction on photovoltaic performance was studied by varying the defect density from 10^10^ to 10^20^ cm^–2^, keeping all other device parameters constant. The results are shown in Figure 5. When the interface defect density is low at 10^10^ cm^–2^, the device yields its best performance with *V*_oc_ = 1.248 V, *J*_sc_ = 24.66 mA/cm^2^, FF = 89.42%, and PCE = 27.52%. The device retains stable performance up to 10^11^ cm^–2^, indicating that a small number of interface defects does not significantly affect hole extraction at the rear contact. As the defect density increases to 10^12^ cm^–2^, a noticeable drop in *V*_oc_ to 1.23 V and PCE to 27.17% is observed, signalling the onset of recombination losses at the rear junction. The degradation becomes more pronounced between 10^12^ and 10^14^ cm^–2^, where *V*_oc_ drops from 1.23 to 1.16 V, *J*_sc_ decreases from 24.61 to 22.90 mA/cm^2^, FF falls from 89.30 to 86.88%, and PCE reduces from 27.17 to 23.16%, corresponding to an absolute efficiency loss of more than 4% over just two decades of defect density. At defect densities beyond 10^15^ cm^–2^, the device performance stabilizes at *V*_oc_ = 1.15 V, *J*_sc_ = 21.81 mA/cm^2^, FF = 85.76%, and PCE = 21.52%, with no further change observed up to 10^20^ cm^–2^. This plateau indicates that recombination at the rear interface fully controls the device output at these high defect levels. Based on these observations, maintaining the Cu_2_O/Cs_2_AgBiI_6_ interface defect density below 10^12^ cm^–2^ is critical to achieving a PCE above 27%. This underlines the importance of careful interface passivation during device fabrication to suppress recombination at the rear junction.

**Figure 5:**
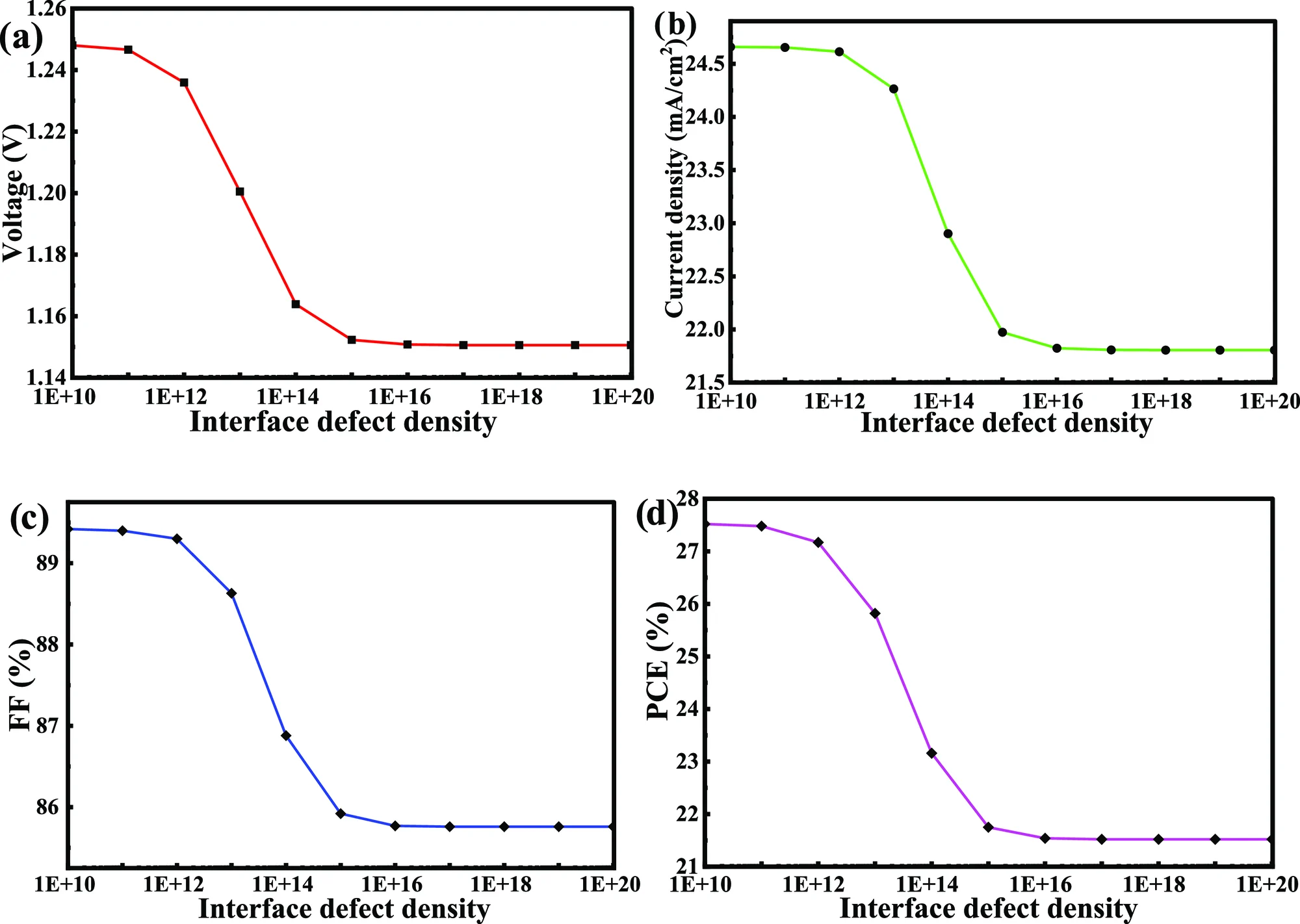
Effect of interface defect density (a) *V*_oc_, (b) *J*_sc_, (c) FF (%), and (d) PCE (%) on the photovoltaic performance of Cu_2_O/Cs_2_AgBiI_6_ PSCs.

### 3.6 Role of perovskite/LBSO Interface Defects in Determining Device Performance

The effect of interface defect density at the Cs_2_AgBiI_6_/LBSO junction was systematically investigated over the range of 10^10^ to 10^20^ cm^–2^, with all other device parameters fixed at their optimized values. The extracted photovoltaic parameters are presented in Figure 6. At the lowest defect density (10^10^ cm^–2^), the device achieves maximum performance with *V*_oc_ = 1.248 V, *J*_sc_ = 24.66 mA/cm^2^, FF = 89.42%, and PCE = 27.52%. As the defect density increases, a pronounced and continuous reduction in *V*_oc_ is observed, decreasing from 1.248 to 0.8206 V across the studied range, corresponding to a total voltage loss of 0.43 V. This behaviour indicates a strong increase in non-radiative recombination at the ETL/absorber interface, which reduces quasi-Fermi level (FL) splitting and limits the achievable *V*_oc_. The *J*_sc_ remains nearly constant (24.66 mA/cm^2^) up to approximately 10^14^ cm^–2^, confirming that carrier generation and initial collection are not significantly affected at low interface defect densities. However, beyond 10^15^ cm^–2^, *J*_sc_ shows a gradual but consistent decline, reaching 24.16 mA/cm^2^ at 10^20^ cm^–2^. This indicates that at high defect densities, recombination losses extend beyond voltage reduction and begin to impact carrier collection efficiency. Similarly, the FF decreases progressively from 89.42 to 84.91%, reflecting increased recombination current and deviation from ideal diode behaviour. As a combined result of these effects, the PCE decreases sharply from 27.52 to 16.84%, with the initial degradation dominated by *V*_oc_ loss and further reduction reinforced by FF and *J*_sc_ deterioration at higher defect densities. Overall, the Cs_2_AgBiI_6_ /LBSO interface is identified as a recombination-sensitive region, with *V*_oc_ being the most affected parameter, followed by FF and *J*_sc_ at elevated defect densities. A practical defect density threshold of ∼10^12^ cm^–2^ is evident, below which the device maintains PCE above 26% with minimal voltage loss. These findings highlight the critical importance of interface defect suppression through targeted strategies such as interfacial engineering to achieve high-efficiency device performance.

**Figure 6:**
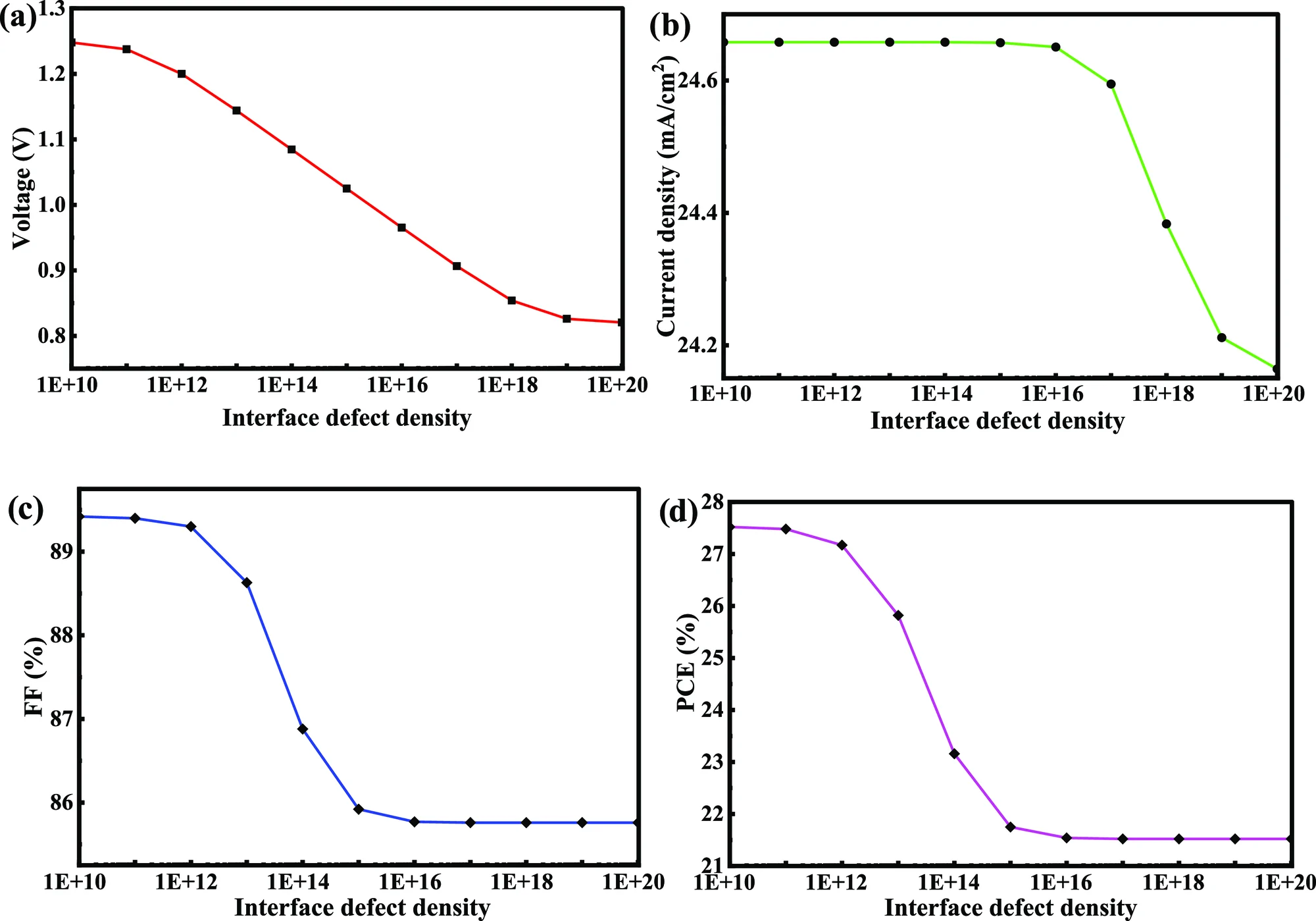
Changes in the photovoltaic characteristics of the Cs_2_AgBiI_6_/LBSO solar cell, namely (a) *V*_oc_, (b) *J*_sc_, (c) FF (%), and (d) PCE (%), as a function of interface defect density (DD).

### 3.7 Impact of Bulk Defect Density on Cs_2_AgBiI_6_ Double PSC Performance

To understand how crystallographic imperfections within the Cs_2_AgBiI_6_ absorber layer affect overall device behaviour, the bulk defect density was varied across a wide range from 10^13^ to 10^20^ cm^–3^ and the corresponding changes in device performance were recorded, with results shown in Figure 7. At a low defect density of 10^13^ cm^–3^, the device exhibits its highest performance, with a PCE of 28.79%, *V*_oc_ of 1.29 V, *J*_sc_ of 24.71 mA/cm^2^, and an FF of 89.72%. These results indicate that the absorber layer can achieve near-optimal performance when the bulk material quality is nearly defect-free. At the standard optimized condition of 10^14^ cm^–3^, performance remains strong at PCE = 27.52%, *V*_oc_ = 1.248 V, *J*_sc_ = 24.66 mA/cm^2^, and FF = 89.42%, confirming that a moderate trap concentration at this level does not significantly compromise carrier collection or voltage output. What makes the bulk defect response particularly striking and physically distinct from what was observed at the device interfaces is that increasing defect density beyond 10^14^ cm^–3^ triggers a simultaneous and unrelenting decline in every performance parameter, with no parameter stabilizing at any point across the sweep. As defect density climbs from 10^15^ to 10^17^ cm^–3^, the degradation accelerates visibly: *V*_oc_ falls from 1.19 to 1.07 V, *J*_sc_ drops sharply from 24.21 to 17.54 mA/cm^2^, and FF deteriorates from 87.98% to just 70.97%, reflecting a situation where defect states distributed throughout the absorber bulk are intercepting photogenerated carriers long before they can reach the transport layers. At very high defect concentrations approaching 10^20^ cm^–3^, the device performance deteriorates substantially *V*_oc_ collapses to 0.6393 V, *J*_sc_ shrinks to just 5.69 mA/cm^2^, FF drops to 53.36%, and PCE falls to only 1.94% an efficiency loss of over 26% absolute from the peak value, driven by a carrier lifetime so short that most photogenerated electrons and holes recombine within the absorber bulk before contributing any useful current. This continuously worsening trend, clearly visible across all four panels in Figure 7 and conspicuously absent of any saturation behaviour, sets bulk defect degradation apart from interface-driven losses and highlights a fundamental physical difference: while interface traps affect only carriers near the junction boundaries, bulk traps penalize every carrier generated anywhere within the 1200 nm absorber volume. Practically speaking, these results send a clear message keeping the bulk defect density at or below 10^14^ cm^–3^ is essential for maintaining a PCE above 27%, and reducing the bulk defect density toward 10^13^ cm^–3^ could increase the efficiency close to 29% by minimizing non-radiative recombination losses and moving the device closer to the Shockley-Queisser limit for a 1.6 eV absorber. Achieving this performance requires high-quality crystalline films through optimized deposition, controlled annealing, and effective bulk and interface defect passivation.

**Figure 7:**
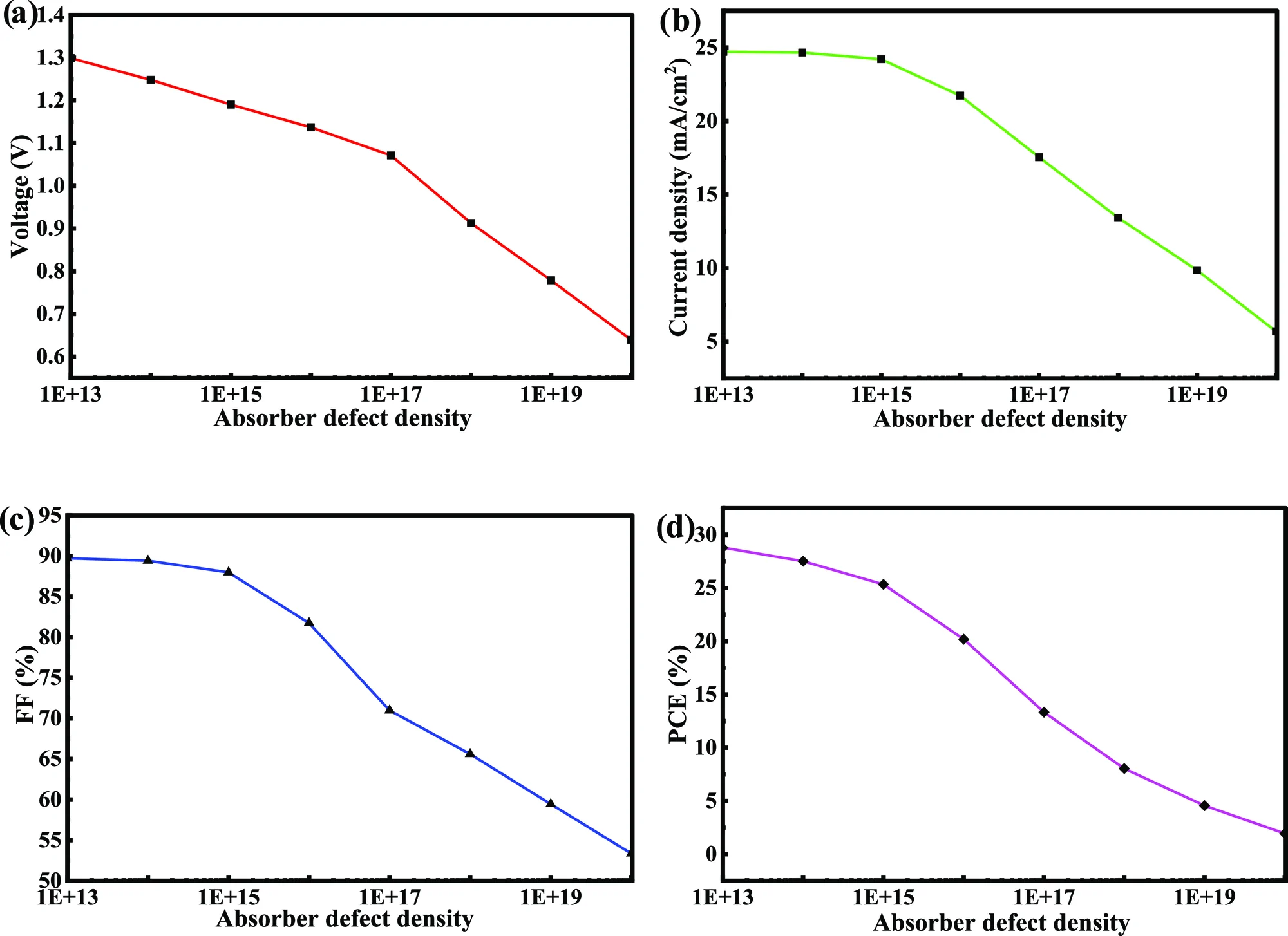
Effect of absorber bulk defect density on (a) *V*_oc_, (b) *J*_sc_, (c) FF (%), and (d) PCE (%) of the Cs_2_AgBiI_6_ PSCs.

The value of 1 × 10^14^ cm^–3^ represents the optimized low defect simulation condition and should not be interpreted as the experimentally achieved defect density of Cs_2_AgBiI_6_ films.

### 3.8 Hole Transport Layer Material Screening and Thickness-Dependent Device Behaviour

HTL candidates Cu_2_O, CBTS, CuSCN, CuI, Spiro-MeOTAD, and PEDOT: PSS were systematically screened across thicknesses of 10 to 100 nm in the FTO/LBSO/Cs_2_AgBiI_6_ /HTL/Au device structure, with the evolution of *V*_oc_, *J*_sc_, FF (%), and PCE (%) presented in Figure 8, A universal trend observed across all six materials is rapid performance saturation beyond 30 nm, where all four figures of merit stabilize into thickness-independent plateaus extending through 100 nm, confirming that once a sufficient HTL thickness is achieved to establish a complete depletion region and block electron back-injection, further increases in layer thickness produce no measurable improvement in device output. Cu_2_O and CBTS are partial exceptions to this immediate saturation, both exhibiting a gradual performance rise between 10 and 30 nm driven by incomplete rear-contact passivation at ultrathin thicknesses, before fully stabilizing at 30 nm. Cu_2_O exhibits the best overall performance, achieving a stabilized PCE of 27.52% with *V*_oc_ = 1.248 V, *J*_sc_ = 24.66 mA/cm^2^, and FF = 89.42%. This improved performance is associated with a favorable valence band alignment at the Cu_2_O/Cs_2_AgBiI_6_ interface, which facilitates efficient hole extraction and reduces interfacial recombination losses. As a result, Cu_2_O delivers higher *V*_oc_ and overall efficiency compared to the other HTL materials. CBTS ranks second with PCE = 27.30%, *V*_oc_ = 1.242 V, *J*_sc_ = 24.63 mA/cm^2^, and FF = 89.17% the highest fill factor in the entire study indicating strong hole extraction capability, though its marginally lower *V*_oc_ compared to Cu_2_O reflects a slightly less favorable rear interface energetics that caps its overall efficiency 0.22% below the best performer. CuSCN and CuI exhibit relatively lower device performance compared to Cu_2_O and CBTS. CuSCN achieves a PCE of ∼23% with *V*_oc_ = 1.20 V, *J*_sc_ = 23 mA/cm^2^, and FF = 84%, while CuI shows a PCE of ∼21% with *V*_oc_ = 1.13 V, *J*_sc_ = 22 mA/cm^2^, and FF = 85%. The reduced performance is associated with lower *V*_oc_ and *J*_sc_, indicating increased recombination losses and less efficient charge extraction at the HTL/absorber interface. These effects are likely related to suboptimal band alignment and interfacial properties compared to the top-performing HTLs. The reduced *V*_oc_ observed for Spiro-MeOTAD suggests increased interfacial recombination, which may be associated with an unfavorable valence band (VB) offset at the HTL/absorber interface, while its known hygroscopic nature may limit its long-term stability in practical applications. PEDOT: PSS performs weakest overall at PCE = 17.31%, *V*_oc_ = 1.19 V, *J*_sc_ = 21.58 mA/cm^2^, and FF = 84.02%, limited by inferior hole extraction efficiency and well-documented moisture-induced degradation under operational conditions.

**Figure 8:**
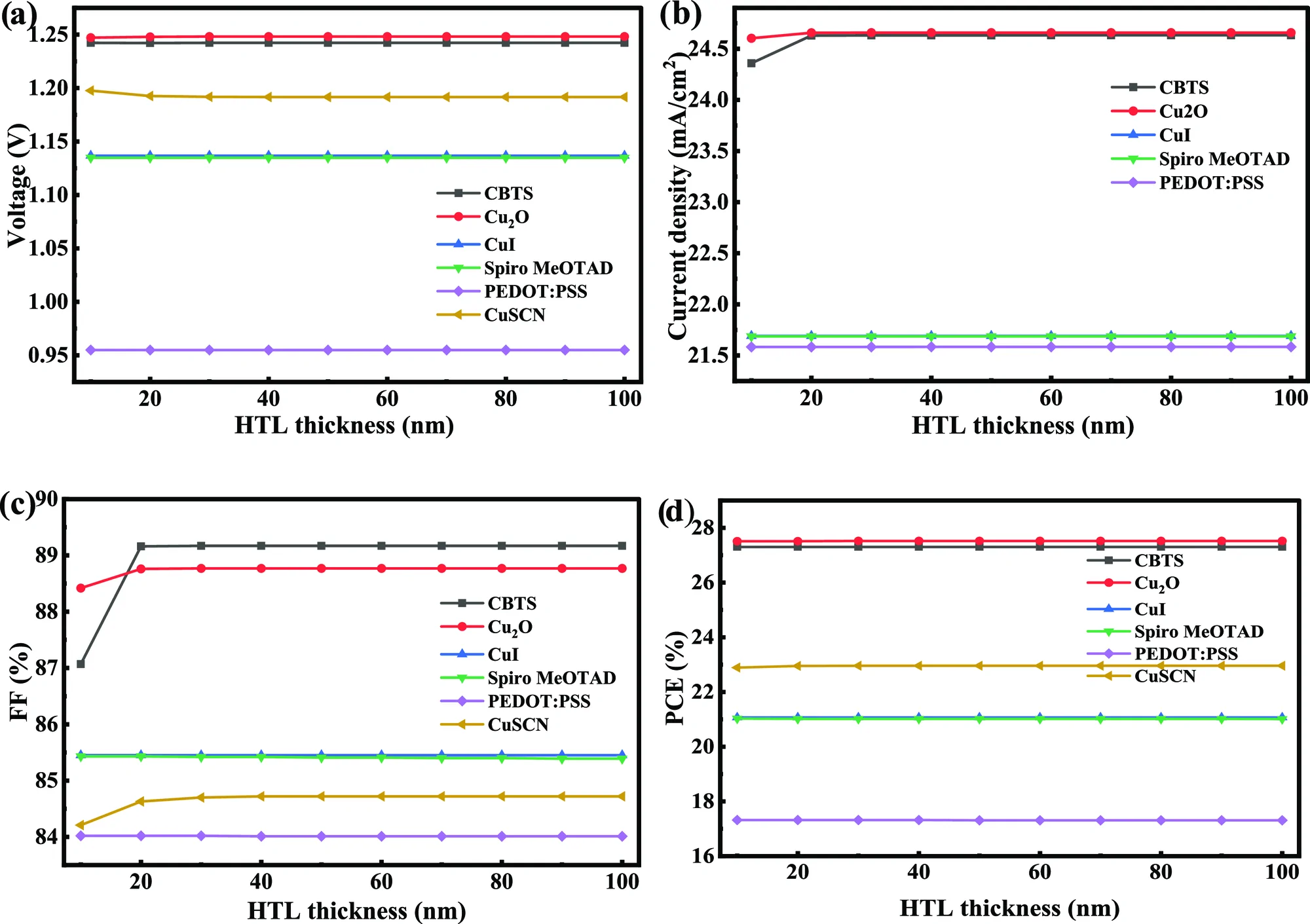
Device performance (a) *V*_oc_, (b) *J*_sc_, (c) FF (%), and (d) PCE (%) on the different HTL materials.

These results identify Cu_2_O as the optimal HTL for this device architecture, delivering the highest PCE of 27.52% and *V*_oc_ of 1.248 V with full performance saturation achieved at just 30-40 nm minimizing material consumption while maximizing device output, and establishing Cu_2_O as the most practical and efficient rear contact candidate for Cs_2_AgBiI_6_ lead-free double PSCs.

### 3.9 Electron Transport Layer Material Screening and Thickness-Dependent Device Behaviour

LBSO, CdS, and ZnS were evaluated as ETL candidates across 10 to 100 nm in the FTO/ETL/Cs_2_AgBiI_6_/Cu_2_O/Au device, with results shown in Figure 9. The three materials exhibit distinctly different thickness-dependent behaviours, providing clear insight into their suitability for this absorber system. LBSO delivers the best performance *V*_oc_ = 1.248 V, *J*_sc_ = 24.66 mA/cm^2^, FF = 89.42%, and PCE = 27.52% with no meaningful variation across the entire 10 to 100 nm range. This thickness-independent stability reflects strong conduction band alignment with Cs_2_AgBiI_6_, ensuring efficient electron extraction with minimal front interface recombination. From a fabrication perspective, this removes the need for precise thickness control, which is a practical advantage. CdS stabilizes quickly beyond 20 nm but delivers considerably lower performance at *V*_oc_ = 1.24 V, *J*_sc_ = 24.68 mA/cm^2^, FF = 64.87%, and PCE = 19.91%. Its poor fill factor relative to LBSO is the key limiting factor, caused by a less favorable conduction band offset that introduces series resistance losses regardless of thickness. ZnS is strongly thickness-dependent. At 10 nm, performance collapses to PCE = 3.40%, *J*_sc_ = 6.08 mA/cm^2^, and FF = 44.76%, indicating incomplete FTO surface coverage and open recombination pathways. Performance recovers progressively with increasing thickness 12.38% at 20 nm, 17.17% at 30 nm, 23.16% at 40 nm, 24.35% at 50 nm before stabilizing at PCE = 24.39%, *V*_oc_ = 1.24 V, *J*_sc_ = 24.54 mA/cm^2^, and FF = 79.61% beyond 60 nm. Even at saturation, ZnS remains 3% below LBSO in PCE and nearly 10% lower in FF a fundamental limitation imposed by its wider bandgap and suboptimal conduction band position that thickness adjustment alone cannot resolve. LBSO is identified as the optimal ETL, combining the highest PCE of 27.52%, higher FF of 89.42%, and thickness-independent performance from just 10 nm making it the most practical and efficient electron transport material for Cs_2_AgBiI_6_ lead-free double perovskite photovoltaics.

**Figure 9:**
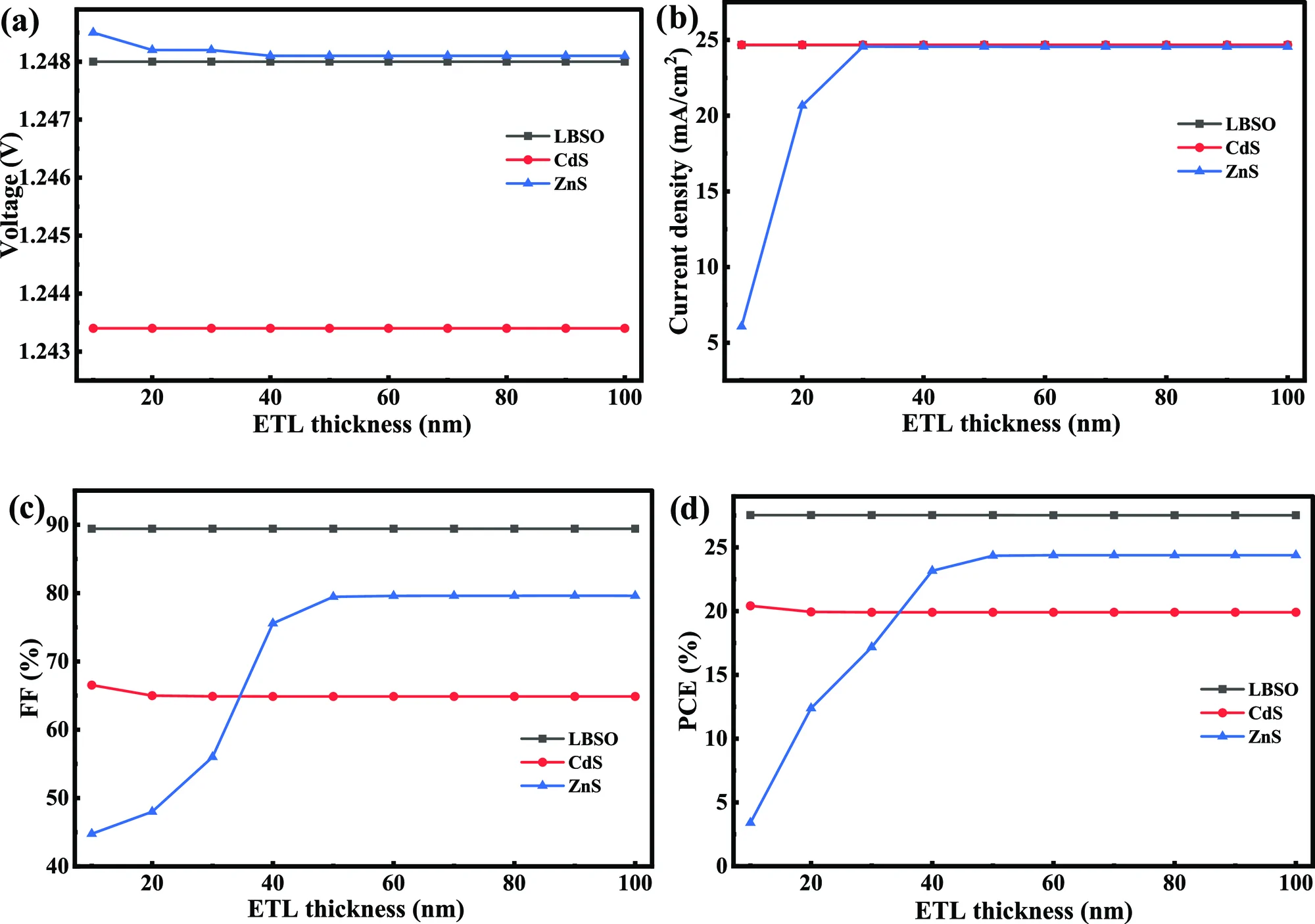
Device performance (a) *V*_oc_, (b) *J*_sc_, (c) FF (%), and (d) PCE (%) on the different ETL materials.

### 3.10 Effect of Absorber Thickness on Device Performance

The influence of Cs_2_AgBiI_6_ absorber thickness on photovoltaic performance was investigated across a range of 100 to 1200 nm in the optimized FTO/LBSO/Cs_2_AgBiI_6_/Cu_2_O/Au configuration, with the resulting trends in *V*_oc_, *J*_sc_, FF (%), and PCE (%) presented in Figure 10. The four parameters exhibit distinctly contrasting monotonic trends with increasing absorber thickness: *J*_sc_ and PCE rise continuously and progressively throughout the entire thickness range, while *V*_oc_ and FF decline steadily, a behaviour that suggests competing effects of enhanced optical absorption and increased recombination losses as the absorber volume grows.

**Figure 10:**
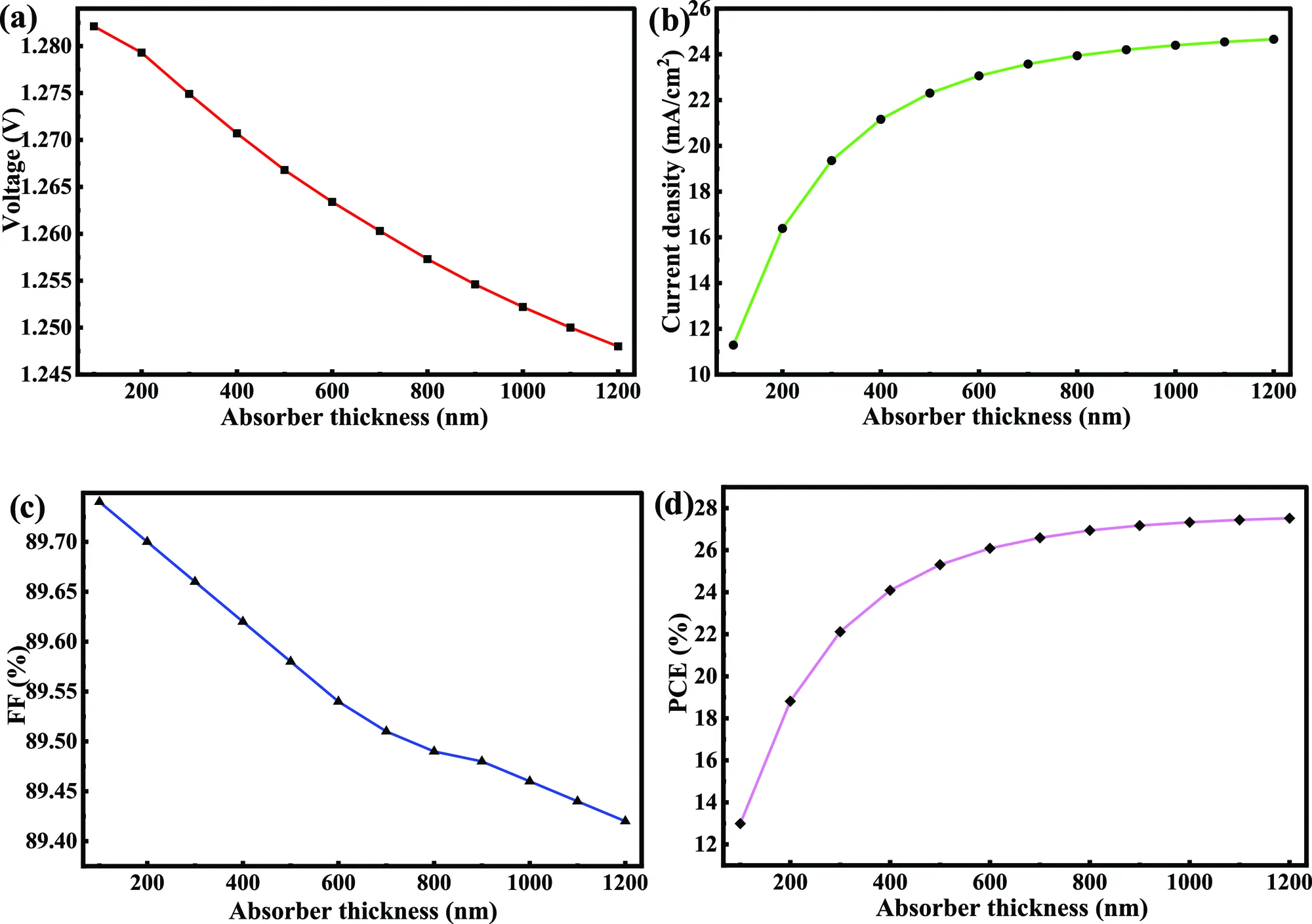
PSC device performance parameters (a) *V*_oc_, (b) *J*_sc_, (c) FF (%), and (d) PCE (%) on absorber thickness.

At the minimum thickness of 100 nm, the device delivers PCE = 12.99%, *V*_oc_ = 1.28 V, *J*_sc_ = 11.29 mA/cm^2^, and FF = 89.74%, a severely current-limited response arising from insufficient absorber volume to harvest a meaningful fraction of the incident solar spectrum, with most long-wavelength photons transmitting through the thin absorber layer without generating electron-hole pairs. As thickness increases from 100 to 600 nm, *J*_sc_ rises sharply from 11.29 to 23.06 mA/cm^2^ and PCE climbs steeply from 12.99 to 26.09%, reflecting the rapid expansion of the optical absorption volume that progressively intercepts a larger portion of the AM1.5G photon flux. Beyond 600 nm the rate of improvement decelerates noticeably, with *J*_sc_ and PCE gains becoming progressively smaller with each additional 100 nm increment from 26.09% at 600 nm to 26.94% at 800 nm and 27.33% at 1000 nm as the absorber approaches a saturation in light absorption and the incremental photon harvesting benefit of additional thickness diminishes. The opposing trend in *V*_oc_ declining continuously from 1.2821 V at 100 nm to 1.248 V at 1200 nm reflects the growing contribution of bulk trap-assisted recombination as the absorber volume expands, which may increase recombination losses and reduce the effective carrier separation. FF follows a parallel but comparatively modest decline from 89.74% at 100 nm to 89.42% at 1200 nm, a total drop of only 0.32% absolute across the entire thickness range indicating that series resistance and carrier collection losses introduced by the thicker absorber remain well controlled throughout, likely owing to the strong built-in field maintained by the LBSO/Cu_2_O carrier-selective contact stack.

The maximum PCE is observed at 1200 nm, where PCE reaches its peak value of 27.52% with *J*_sc_ = 24.66 mA/cm^2^, *V*_oc_ = 1.248 V and FF = 89.42%, a point at which the photocurrent gain from additional light absorption outweighs the modest voltage and fill factor penalties from increased bulk recombination. The optimized absorber thickness of 1200 nm represents the best simulated device performance under the adopted conditions and should be regarded as a theoretical design guideline for future experimental investigations.

### 3.11 Effect of Operating Temperature on Device Performance

The thermal stability of the optimized FTO/LBSO/Cs_2_AgBiI_6_/Cu_2_O/Au PSC was examined across 300 K to 500 K, with results presented in Figure 11. At 300 K, the device delivers its peak performance of *V*_oc_ = 1.248 V, *J*_sc_ = 24.66 mA/cm^2^, FF = 89.42%, and PCE = 27.52%.

**Figure 11:**
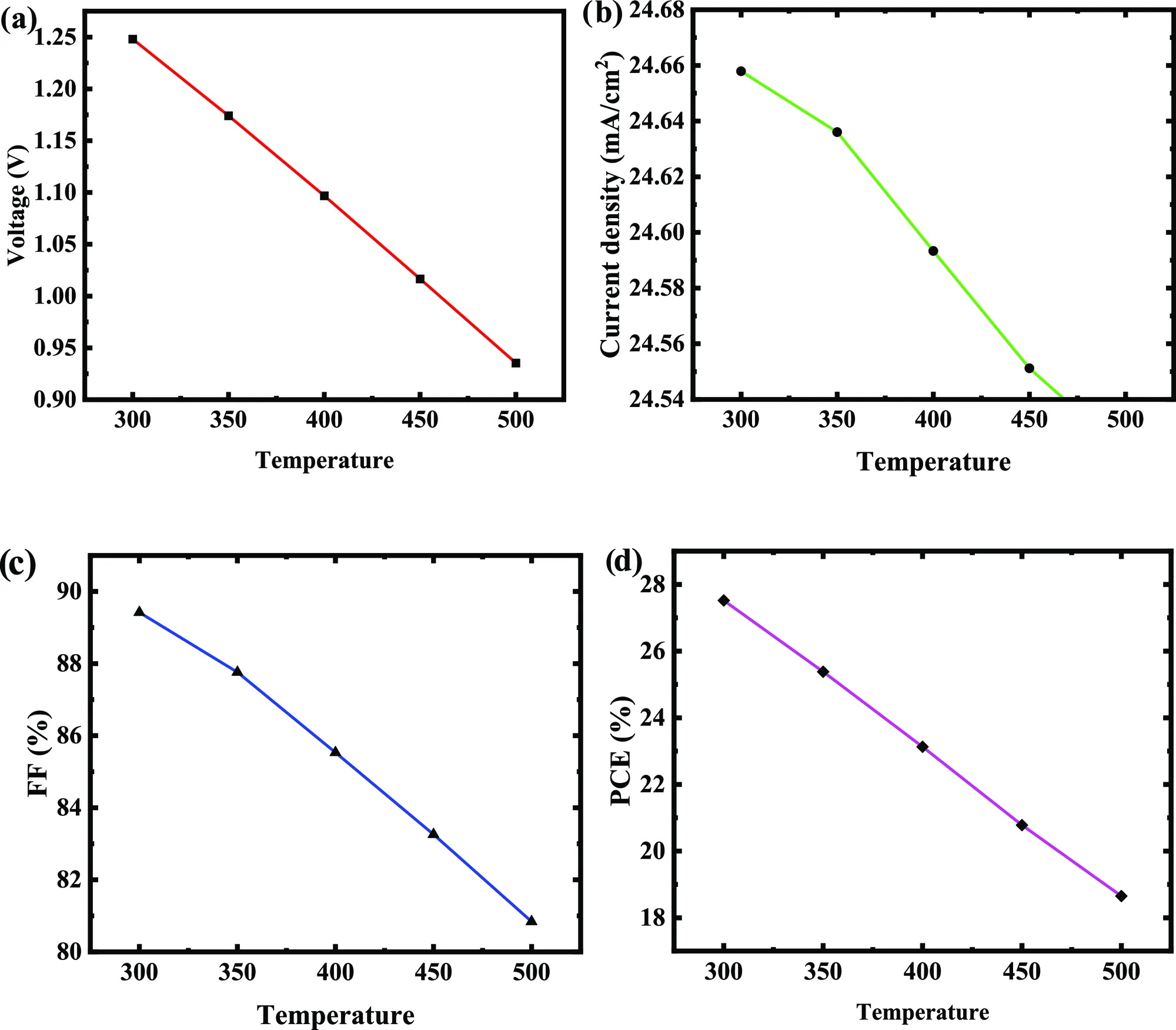
PSC device performance parameters (a) *V*_oc_, (b) *J*_sc_, (c) FF (%), and (d) PCE (%) on various temperature.

As temperature rises, all four parameters decline monotonically. *V*_oc_ suffers the most severe degradation reducing by 0.3126 V across the full range a well-established behaviour in PSCs where elevated thermal energy amplifies intrinsic carrier generation, accelerates non-radiative recombination at grain boundaries and interfaces, and erodes quasi-Fermi level splitting. FF declines by 8.58% absolute, consistent with the thermally induced increase in dark recombination current that degrades the diode quality factor in perovskite devices. *J*_sc_ remains nearly invariant with a reduction of only 0.14 mA/cm^2^, reflecting the stable optical absorption characteristics of the all-inorganic Cs_2_AgBiI_6_ absorber under thermal stress a notable advantage over organic-cation perovskites whose photocurrent is more sensitive to thermally driven phase instability. PCE drops from 27.52% at 300 K to 18.65% at 500 K, with *V*_oc_ and FF degradation as the dominant loss mechanisms. The device retains PCE above 25% up to 350 K, encompassing typical real-world operating temperatures for rooftop and ground-mounted photovoltaic installations. The all-inorganic Cs_2_AgBiI_6_ composition provides inherently superior thermal resilience compared to methylammonium or formamidinium-based perovskite cells, whose organic A-site cations undergo thermally activated decomposition under prolonged elevated temperature exposure, further validating the practical suitability of this lead-free double perovskite architecture for outdoor photovoltaic deployment.

### 3.12 *J*–*V* Characteristics of the optimized device

The *J*–*V* curve of the optimized FTO/LBSO/Cs_2_AgBiI_6_/Cu_2_O/Au device under AM1.5G illumination is shown in Figure 12, giving a short-circuit current density of 24.66 mA/cm^2^ and an open-circuit voltage of 1.248 V. Between 0 and 1.0 V, the current density stays remarkably flat at around 24.66 mA/cm^2^, which tells us that photogenerated carriers are being collected efficiently with very little recombination taking place across this voltage range. As the voltage is pushed beyond 1.0 V, the current begins to fall more rapidly, with a steep drop occurring between 1.18 and 1.26 V before crossing zero at the open-circuit condition. The curve that comes out is almost a rectangle, with a wide flat area followed by a sharp turn close to Voc. This visually shows why the device has such a high fill factor of 89.42%. This is because of low series resistance and well-controlled recombination at both the front and rear junctions. Together with the high *J*_sc_ and *V*_oc_ values, these factors show that the LBSO/Cu_2_O contact combination gives the Cs_2_AgBiI_6_ absorber the best photovoltaic performance, with a peak PCE of 27.52%. The reported PCE and FF represent the optimized performance predicted under the ideal assumptions of the SCAPS-1D model. In practical devices, additional losses such as series resistance, shunt leakage, contact resistance, and fabrication-related imperfections are expected to reduce the achievable performance.

**Figure 12:**
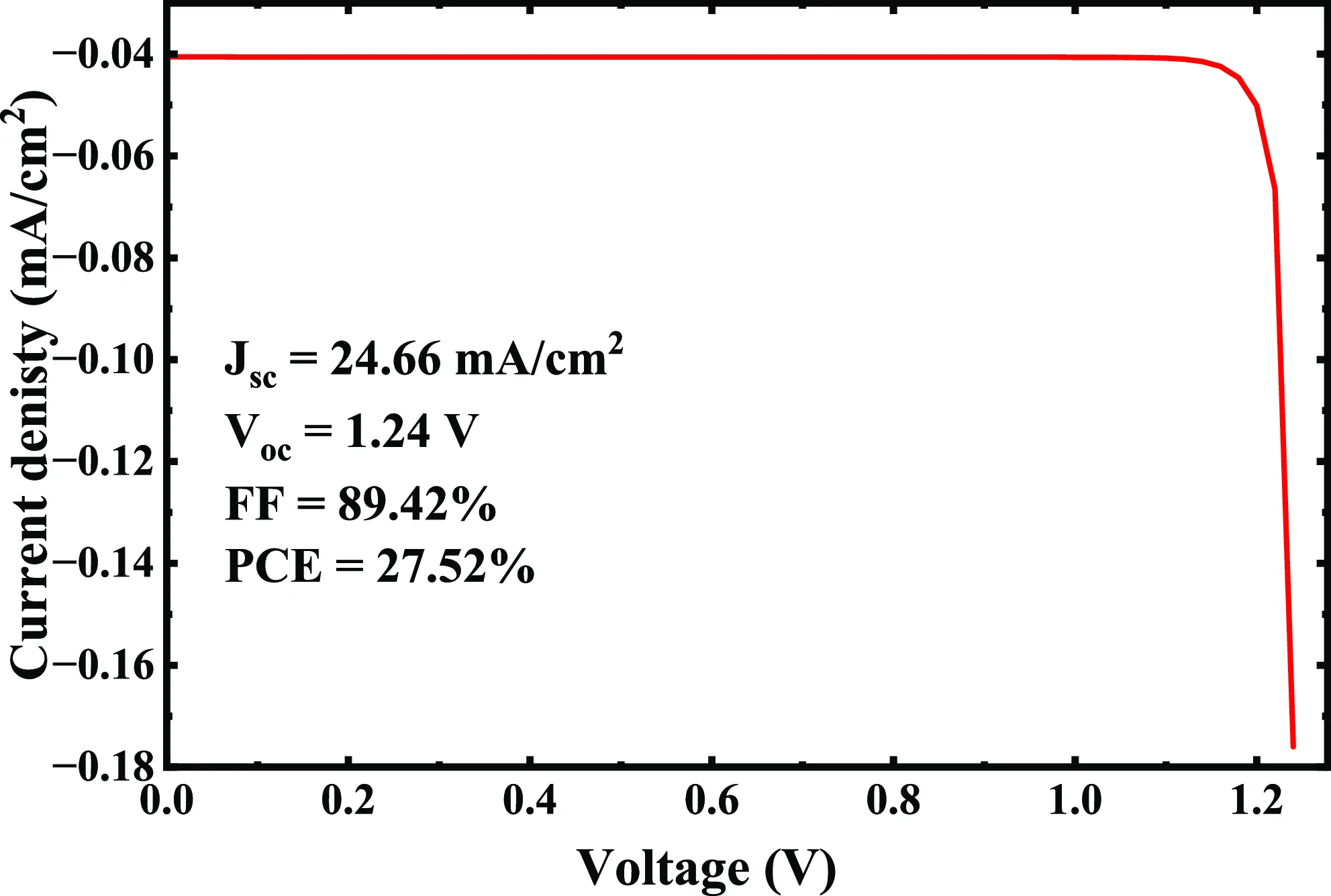
*J*–*V* Characteristics of perovskite solar cell.

### 3.13 Performance Comparison of Cs_2_AgBiI_6_ on SCAPS-1D Simulations

Table 4 benchmarks the optimized FTO/LBSO/Cs_2_AgBiI_6_/Cu_2_O/Au device against existing SCAPS-1D literature on Cs_2_AgBiI_6_. The computational investigation of Cs_2_AgBiI_6_ as a photovoltaic absorber has attracted considerable attention, with various research groups employing different transport layer combinations to maximize device performance. Simulated efficiencies across these studies have generally fallen between 21 and 24%, with incremental gains attributed to improved contact selection, better band alignment strategies, and reduced interfacial defect densities. The present study contributes to this body of work by demonstrating that the LBSO/Cu_2_O contact pair offers a favorable charge extraction environment for this double perovskite absorber, yielding a PCE that compares competitively with and in several cases surpasses previously reported values. The simulated PCE is higher than previous SCAPS-1D results, while experimental devices show much lower performance due to defects, interface losses, and fabrication limitations. Thus, the 27.52% PCE represents an optimized theoretical prediction value.

**Table 4:** SCAPS-1D Simulated Photovoltaic Performance of Cs_2_AgBiI_6_ Solar Cells Compared with Prior Literature

**PSC device architecture**	***V*_oc_ (V)**	***J*_sc_** (mA cm^**–2**^ **)**	**fill factor (FF, %)**	**PCE (%)**	**refs**
FTO/TiO_2_/ Cs_2_AgBiI_6_/Cu_2_O/Au	0.960	24.98	83.16	19.94	^29^
FTO/TiO_2_/ Cs_2_AgBiI_6_/SnS/Au	0.940	22.52	86.85	18.37	^30^
ITO/TiO_2_/Cs_2_AgBiI_6_/CBTS/Au	1.180	22.40	84.50	22.30	^31^
ITO/ZnO/Cs_2_AgBiI_6_/CBTS/Au	0.931	25.42	86.95	21.55	^32^
FTO/SnO_2_/Cs_2_AgBiI_6_/CBTS/Au	1.142	23.45	85.10	22.80	^33^
FTO/WS_2_/Cs_2_AgBiI_6_/CuI/Au	1.105	23.20	84.30	21.60	^34^
ITO/IGZO/Cs_2_AgBiI_6_/Cu_2_O/Au	1.165	23.60	84.80	23.30	^35^
FTO/ZnOS/Cs_2_AgBiI_6_/CZTS/Au	1.150	23.10	83.20	24.11	^36^
FTO/LBSO/Cs_2_AgBiI_6_/CBTS/Au	1.242	24.63	89.17	27.30	this work
FTO/LBSO/Cs_2_AgBiI_6_ /Cu_2_O/Au	1.248	24.66	89.42	27.52	this work

## 4 Conclusion

In this study, lead-free Cs_2_AgBiI_6_ double PSCs were studied using DFT calculations alongside SCAPS-1D device simulations. The DFT results confirmed that the material has an indirect bandgap of 1.6 eV, where the valence band is shaped by Ag–I hybridized states and the conduction band is governed by Bi p-orbitals. The DFT calculations provided theoretical insight into the electronic characteristics of Cs_2_AgBiI_6_, whereas the SCAPS-1D simulations were independently performed using material parameters adopted from the literature. For the ETL, LBSO gave the best results among the candidates tested, mainly because of its good band alignment with the absorber and its ability to maintain consistent electron extraction across a wide range of thicknesses. On the hole transport side, Cu_2_O performed better than the other candidates, largely due to lower recombination losses at the rear interface that helped preserve the *V*_oc_. The simulations also showed that device efficiency drops considerably when the interface defect density goes beyond 10^12^ cm^–2^ or when the bulk defect density in the absorber exceeds 10^14^ cm^–3^. An absorber thickness of 1200 nm was found to give the right balance between absorbing enough light and collecting the generated carriers effectively. The best device in this study, with the structure FTO/LBSO/Cs_2_AgBiI_6_/Cu_2_O/Au, reached a *V*_oc_ of 1.248 V, *J*_sc_ of 24.66 mA/cm^2^, FF of 89.42%, and a PCE of 27.52% under AM1.5G illumination. These values represent the optimized theoretical performance predicted by SCAPS-1D and serve as a computational reference for future experimental validation. The results provide theoretical guidance for the design and optimization of high-performance lead-free double perovskite solar cells.
